# Supplementary figures and images for: Integrin adhesome axis inhibits the RPM-1 ubiquitin ligase signaling hub to regulate growth cone and axon development
Source: PLoS Genet. 2024 Dec 13;20(12):e1011496. doi: 10.1371/journal.pgen.1011496 (PMC11642917; doi:10.1371/journal.pgen.1011496)

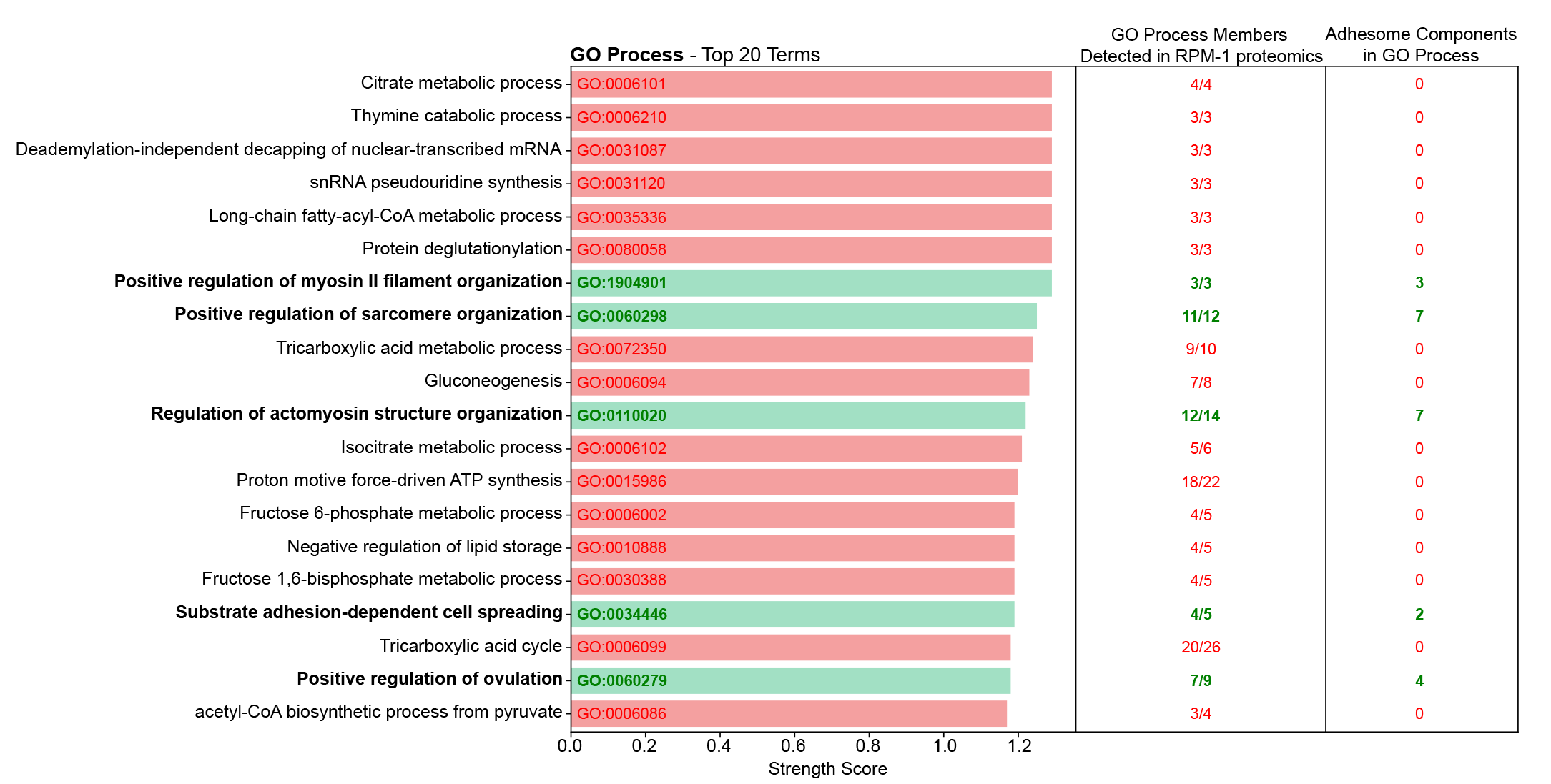

Supplement: S1 Fig — (TIF) [file pgen.1011496.s001.tif]

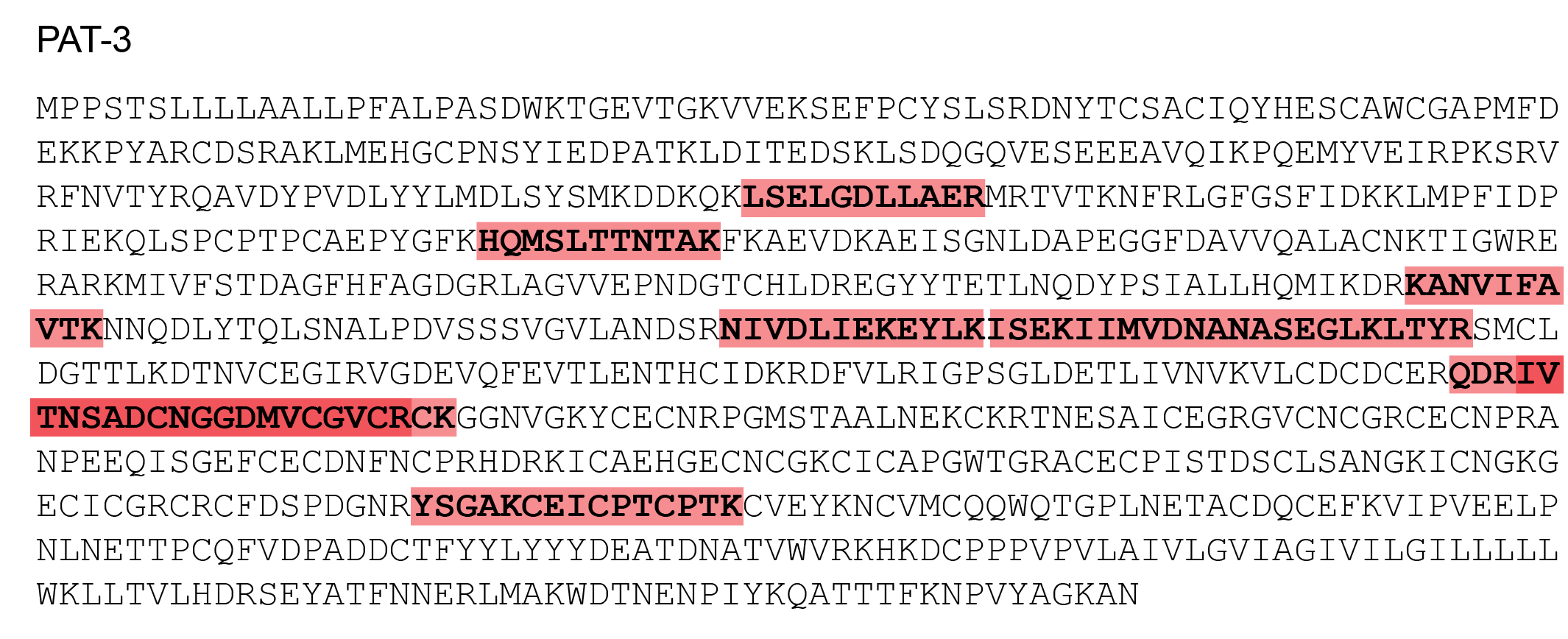

Supplement: S2 Fig — Highlighted are 8 peptides in PAT-3 (red) identified in GS::RPM-1 and GS::RPM-1 LD samples. (TIF) [file pgen.1011496.s002.tif]

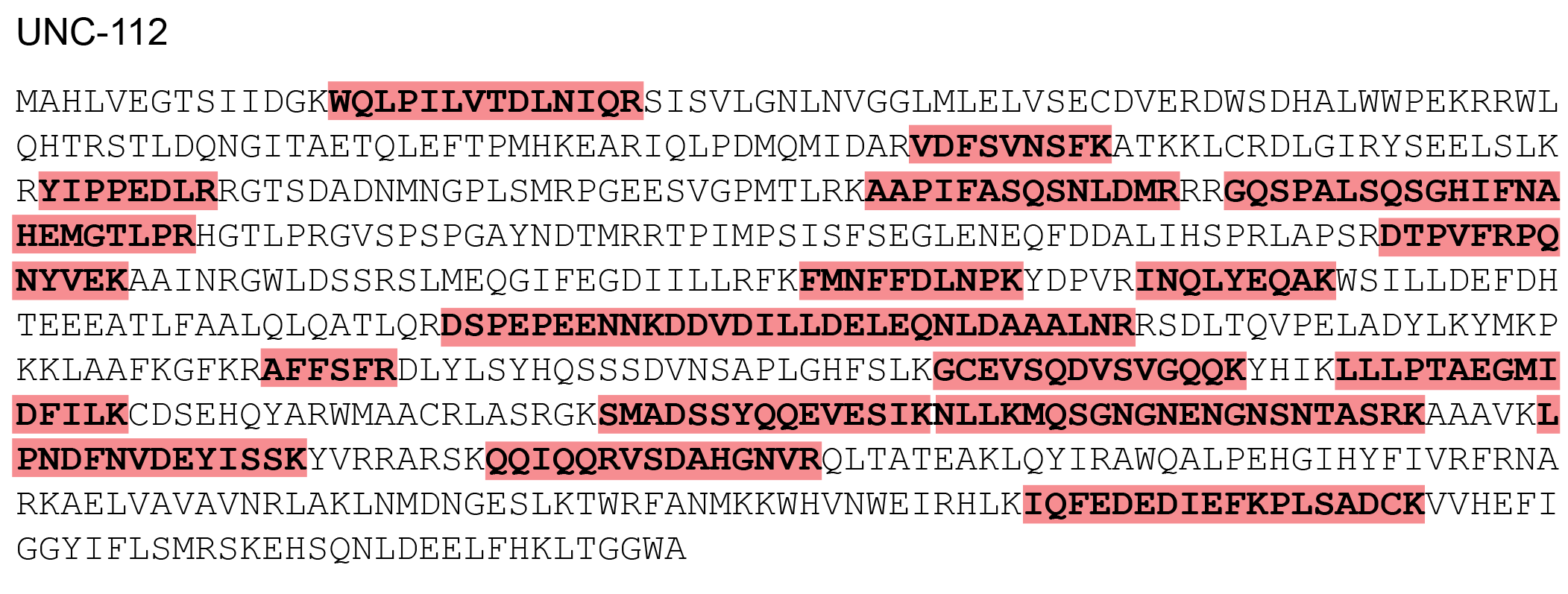

Supplement: S3 Fig — Highlighted are 17 peptides in UNC-112 (red) identified in GS::RPM-1 and GS::RPM-1 LD samples. (TIF) [file pgen.1011496.s003.tif]

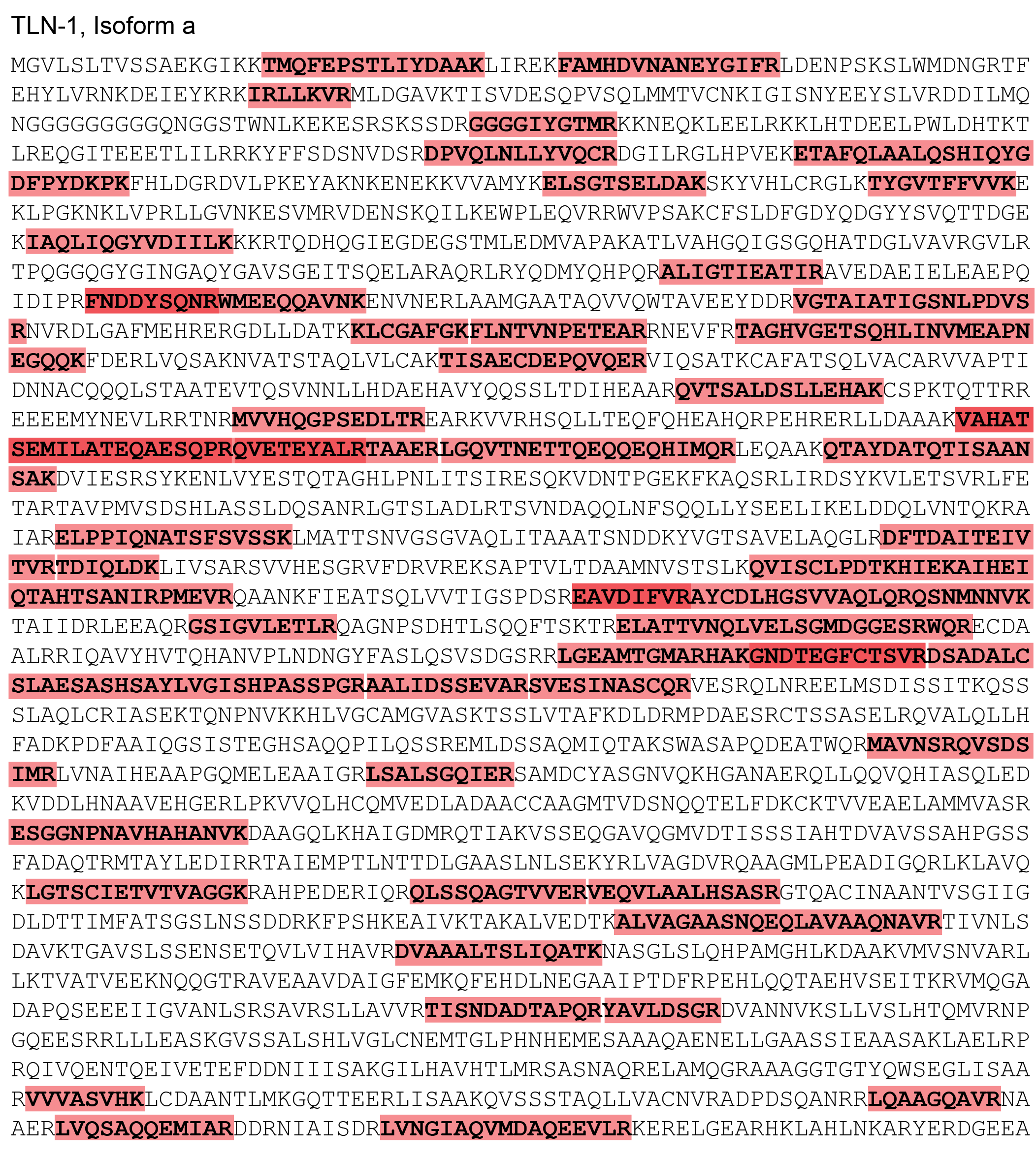

Supplement: S4 Fig — Highlighted are 51 peptides in TLN-1 (red) identified in GS::RPM-1 and GS::RPM-1 LD samples. (TIF) [file pgen.1011496.s004.tif]

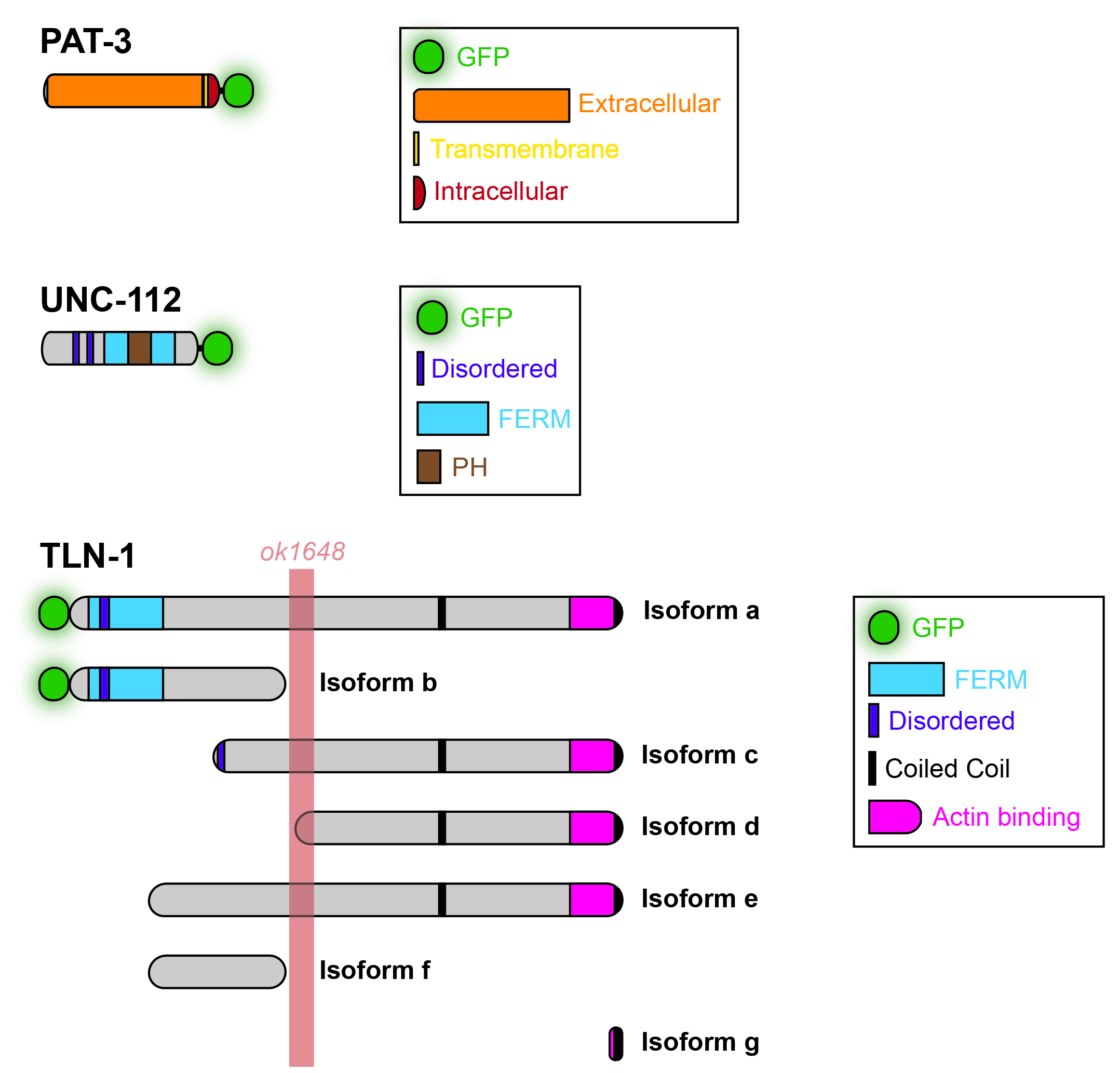

Supplement: S5 Fig — Protein diagrams with annotated protein domains highlight location of CRISPR engineered GFP tag on PAT-3, UNC-112 and TLN-1. Note that only two TLN-1 isoforms, TLN-1 a and b, are tagged with GFP. Also annotated is tln-1 hypomorphic deletion allele ok1648 (red box), which only affects TLN-1 isoforms a, c, d, and e. (TIF) [file pgen.1011496.s005.tif]

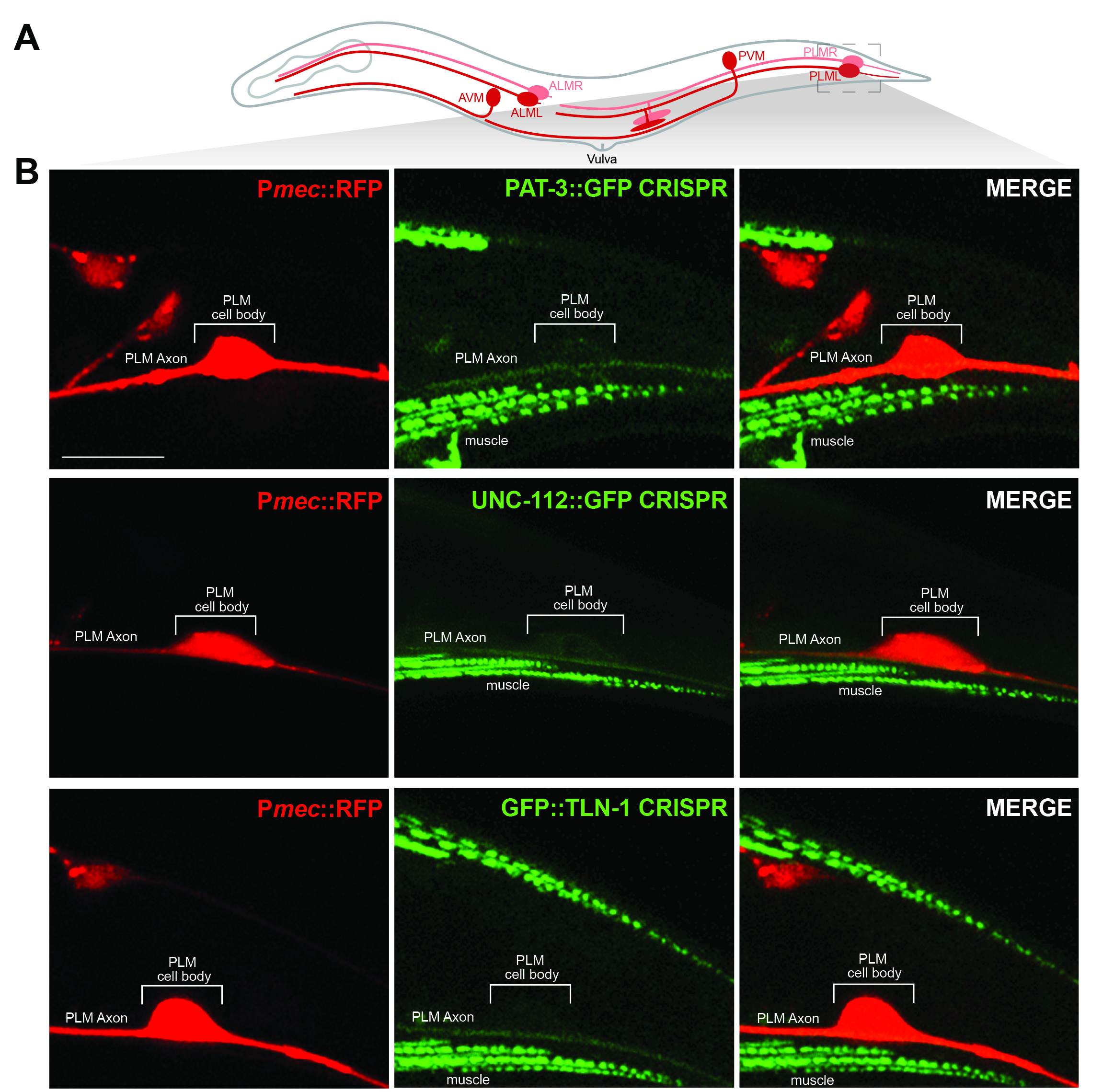

Supplement: S6 Fig — A) Schematic of PLM neurons with imaged regions highlighted (light gray box). B) Representative images showing that PAT-3::GFP, UNC-112::GFP and GFP::TLN-1 are localized to PLM soma at low levels compared to axon localization. PAT-3::GFP and GFP::TLN-1 images acquired at 40x magnification and UNC-112::GFP images acquired at 100x magnification. Scale bar 10μm. (TIF) [file pgen.1011496.s006.tif]

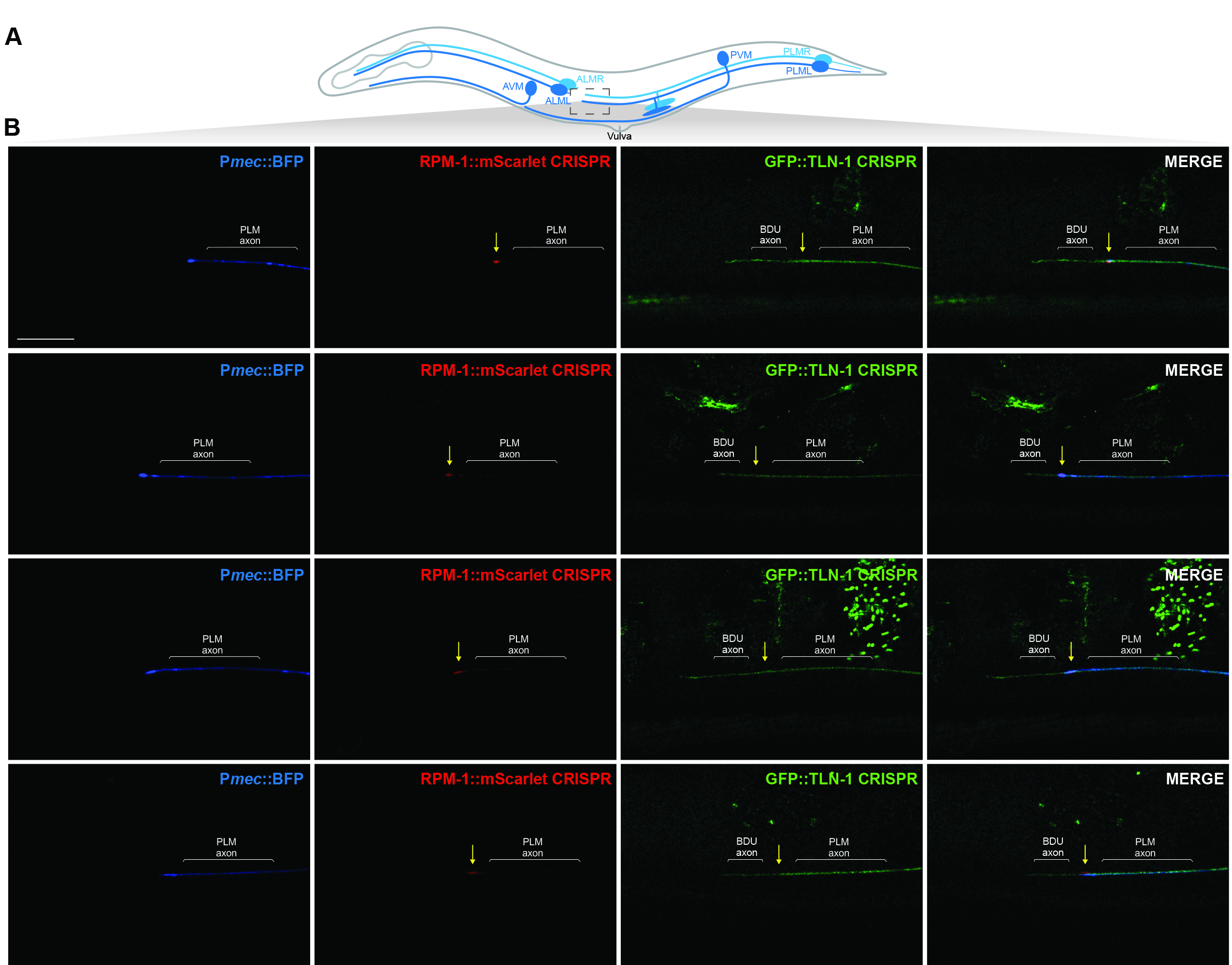

Supplement: S7 Fig — Shown are multiple examples of super-resolution images demonstrating CRISPR engineered GFP::TLN-1 and RPM-1::mScarlet colocalize at terminated axon tips of PLM neurons. PLM axons were visualized using transgenic BFP expressed in mechanosensory neurons (Pmec-17::BFP, bggEx180). Note GFP::TLN-1 expression anterior to the PLM termination site is neurite from adjacent BDU neuron. Scale bar 10μm. (TIF) [file pgen.1011496.s007.tif]
