# Supplementary material for: Integrin adhesome axis inhibits the RPM-1 ubiquitin ligase signaling hub to regulate growth cone and axon development
Source: PLoS Genet. 2024 Dec 13;20(12):e1011496. doi: 10.1371/journal.pgen.1011496 (PMC11642917; doi:10.1371/journal.pgen.1011496)
Supplement: S4 Table. C — (DOCX) [file pgen.1011496.s011.docx]

**Table S4: Transgenic and CRISPR Strains**

| **Figure** | **Strain** | **Genotype** |
| --- | --- | --- |
| Figure 1, 2 | XMN829 | *muIs32* [P_mec-7_::GFP] II; *rpm-1*(*ju44*) *bggIs9* [P_rpm-1_::GS::RPM-1; P_myo-2_::mCherry; pha-1(+)] V |
| Figure 1 | XMN830 | *muIs32* [P_mec-7_::GFP]; *bggIs19* [P_rpm-1_::GS::RPM-1 LD  P_myo-2_::mCherry; pha-1(+)] II; *rpm-1*(*ju44*) V |
| Figure 1, 2 | XMN831 | *muIs32* [P_mec-7_::GFP] II; *rpm-1*(*ju44*) V; *bggIs23* [P_rpm-1_::GS::GFP; P_myo-2_:: mCherry, pha-1(+)] |
| Figure 3, 5 | XMN1242 | *pat-3*(*bgg86* [PAT-3::GFP CRISPR]); *jsIs973* [P_mec-7_::mRFP; unc-119 (+)] III |
| Figure 3, 5, 7 | XMN1231 | *tln-1*(*zh117* [GFP::TLN-1 CRISPR]) I; *jsIs973* [P_mec-7_::mRFP, unc-119 (+)] III |
| Figure 3, 5 | XMN1168 | *jsIs973* [P_mec-7_::mRFP, unc-119(+)] III; *unc-112*(*bgg68* [UNC-112::GFP CRISPR]) V |
| Figure 4 | XMN1450 | *tln-1*(*zh117* [GFP::TLN-1] I; *rpm-1*(*bgg119* [rpm-1::mScarlet CRISPR] V; *bggEx180* (P_mec-17_::mTagBFP2, P_ttx-3_::RFP) |
| Figure 5, 6, 8 | XMN913 | *jsIs973* [P_mec-7_::mRFP, unc-119(+)] III |
| Figure 5 | XMN1167 | *itSi953* [P_mec-18_::*mecDEG*, unc-119(+)] II; *jsIs973* [P_mec-7_::mRFP, unc-119(+)] III |
| Figure 5, 6 | XMN1243 | *itSi953* [P_mec-18_::*mecDEG*, unc-119(+)] II; *pat-3*(*bgg86* [PAT-3::GFP]) *jsIs973* [P_mec-7_::mRFP, unc-119(+)] III |
| Figure 5, 6, 7, 8 | XMN1230 | *tln-1*(*zh117* [GFP::TLN-1 CRISPR]) I; *itSi953* [P_mec-18_::*mecDEG*, unc-119(+)] II; *jsIs973* [P_mec-7_::mRFP, unc-119(+)] III |
| Figure 5, 6 | XMN1359 | *itSi953* [P_mec-18_::*mecDEG*, unc-119(+)] II; *jsIs973* [P_mec-7_::mRFP, unc-119(+)] III; *unc-112*(*bgg68* [UNC-112::GFP CRISPR]) V |
| Figure 5 | XMN1229 | *tln-1*(*ok1648*) I; *jsIs973* [P_mec-7_::mRFP, unc-119(+)] III |
| Figure 6, 8 | XMN1152 | *jsIs973* [P_mec-7_::mRFP, unc-119(+)] III; *rpm-1(ju44)* V |
| Figure 6, 7 | XMN1272 | *itSi953* [P_mec-18_::*mecDEG*, unc-119(+)] II; *jsIs973* [P_mec-7_::mRFP, unc-119(+)] III; *rpm-1(ju44)* V |
| Figure 6 | XMN1366 | *itSi953* [P_mec-18_::*mecDEG*, unc-119(+)] II; *pat-3*(*bgg86* [PAT-3::GFP]) *jsIs973* [P_mec-7_::mRFP, unc-119(+)] III; *rpm-1(ju44)* V |
| Figure 6, 7 | XMN1305 | *rpm-1(ju44)* V; *tln-1*(*zh117* [GFP::TLN-1 CRISPR] I;  *itSi953* [P_mec-18_::*mecDEG*, unc-119(+)] II; *jsIs973* [P_mec-7_::mRFP, unc-119(+)] III |
| Figure 6 | XMN1258 | *itSi953* [P_mec-18_::*mecDEG*, unc-119(+)] II; *jsIs973* [P_mec-7_::mRFP, unc-119(+)] III; *rpm-1 (ju44)*; *unc-112*(*bgg68* [UNC-112::GFP]) V |
| Figure 5 | XMN1412 | *tln-1*(*zh117* [GFP::TLN-1 CRISPR]) I; *itSi953* [P_mec-18_::*mecDEG*, unc-119(+)] II; *jsIs973* [P_mec-7_::mRFP, unc-119(+)] III; *bggEx172* (P_rgef-1_::FLAG::TLN-1, P_rps-27_::NeoR) |

*mecDEG =* ltSi953 [mec-18p::vhhGFP4::zif-1::operon-linker::mKate::tbb-2 3'UTR + Cbr-unc-119(+)] II
