# Supplementary material for: Integrin adhesome axis inhibits the RPM-1 ubiquitin ligase signaling hub to regulate growth cone and axon development
Source: PLoS Genet. 2024 Dec 13;20(12):e1011496. doi: 10.1371/journal.pgen.1011496 (PMC11642917; doi:10.1371/journal.pgen.1011496)
Supplement: S5 Table — (DOCX) [file pgen.1011496.s012.docx]

**Table S5: Primers for genotyping and cloning**

| **Gene** | **Allele/ Construct** | **Primer Sequence** |
| --- | --- | --- |
| *mecDEG* Transgene | *itSi953* | ttTi5605 fwd: 5’ TTTCTCAGTTGTGATACGGTTTTT 3’  Int: 5’ aggaacagaataacagatgatgagc 3’  ttTi5605 rev: 5’ CGCTACTTACCGGAAACCAA 3’ |
| *tln-1* | *ok1648* | ok1648 fwd: 5’ gagccaaatgacgagtaggg 3’  wt rev: 5’ AGAGTCGAACGGATGTTTCG 3’  ok1648 rev: 5’ tctcatggatcgctttttcg 3’ |
| *tln-1* | *zh117* | zh117 fwd: 5’ TCCAGATAAACCGCAAATCC 3’  zh117 Int: 5’ GGCAGACAAACAAAAGAATGG 3’  zh117 rev: 5’ ATCTGGGCGTGGTTTGTTAG 3’ |
| *pat-3* | *bgg86* | bgg86 fwd: 5’ TTATGGCCAAATGGGATACG 3’  bgg86 rev: 5’ TGTGAGTTGTTGGTCGGTGT 3’  bgg86 Int: 5’ GGCAGACAAACAAAAGAATGG 3’ |
| *unc-112* | *bgg68* | wt fwd: 5’ CGAAGCAAAGAACATTCTCAA 3’  bgg68 fwd: 5’ GGCAGACAAACAAAAGAATGG 3’  wt rev: 5’ TTGTACCTACGTTTGCCTAC 3’ |
| *rpm-1* | *ju44* | *ju44* fwd: 5’ CGTGTATGACCTGTAAACGAGAAG 3’  *ju44* rev: 5’ GACATGTTGGAAGAAGATGTTTTG 3’  Digest with AccI |
| *rpm-1* | *bgg119* | bgg119 fwd: 5’ GATGAGCAGTGGCTGGTTAG 3’  bgg119 Int: 5’ ACATGAATTGTGGGGAGAG 3’  bgg119 rev: 5’ CTGGTTTCCACGACTTCACAT 3’ |
