# Supplementary material for: Integrin adhesome axis inhibits the RPM-1 ubiquitin ligase signaling hub to regulate growth cone and axon development
Source: PLoS Genet. 2024 Dec 13;20(12):e1011496. doi: 10.1371/journal.pgen.1011496 (PMC11642917; doi:10.1371/journal.pgen.1011496)
Supplement: S6 Table — (DOCX) [file pgen.1011496.s013.docx]

**Table S6: Injection Conditions**

| **Figure** | **Transgene** | **Injected Strain** | **Injection Mix** |
| --- | --- | --- | --- |
| **Figures 3, 5, 6** | PAT-3::GFP CRISPR (*bgg86*) | **N2** | 4uL Cas9 |
|  |  |  | 5.0uL IDT tracrRNA (0.8ug/uL) |
|  |  |  | 2.8uL IDT crRNA (0.8ug/uL) |
|  |  |  | 1.6uL pRF4 (rol-6 plasmid) |
|  |  |  | 2ug hybrid repair template |
| **Figures 3, 5, 6** | UNC-112::GFP CRISPR (*bgg68*) | **N2** | 5μL tracrRNA (4μg/μL) |
|  |  |  | 0.4μL dpy-10 crRNA (8μg/μL) |
|  |  |  | 0.55μL dpy-10 repair ssODN (500μg/μL) |
|  |  |  | 1μL UNC-112::GFP crRNA (8ug/μL) |
|  |  |  | 6.8μL UNC-112::GFP PCR Template (500ng/μL) |
|  |  |  | 0.5μL KCl (1M) |
|  |  |  | 0.75uL Hepes pH 7.4 (200mM) |
|  |  |  | 4.6μL Nuclease-Free H_2_0 |
|  |  |  | 5μL Cas9 (10μg/μL) |
| **Figure 5** | TLN-1 Rescue  (*bggEx172*) | *tln-1*(*zh117* [GFP::TLN-1]) I; *itSi953* [P_mec-18_::*mecDEG*, *unc-119*(+)] II; *jsIs973* [P_mec-_*_7_*::mRFP, *unc-119*(+)] III | 65ng/μL pBluescript (pBG-49) |
|  |  |  | 10ng/μL P_rps-27_::NeoR (pBG-264) |
|  |  |  | 25ng/μL P_rgef-1_::FLAG::TLN-1 (pBG-GY1100) |
| **Figure 4** | RPM-1::mScarlet CRISPR  (*bgg119*) | **N2** | 4uL Cas9 |
|  |  |  | 5.0uL IDT tracrRNA (0.8ug/uL) |
|  |  |  | 2.8uL IDT crRNA (0.8ug/uL) |
|  |  |  | 1.6uL pRF4 (rol-6 plasmid) |
|  |  |  | 2ug hybrid repair template |
| **Figure 4** | P_mec- 17_::mTagBFP2  (*bggEx180)* | *tln-1(zh117* [GFP::tln-1] I; *rpm-1(bgg119* [rpm-1::mScarlet CRISPR] V | 10ng/uL P_mec-17_::mTagBFP2::let-858 3'UTR (pBG-GY1121) |
|  |  |  | 50ng/uL P_ttx-3_::RFP (pBG-41) |
|  |  |  | 80ng/uL pBluescript (pBG-49) |
