# Supplementary material for: Integrin adhesome axis inhibits the RPM-1 ubiquitin ligase signaling hub to regulate growth cone and axon development
Source: PLoS Genet. 2024 Dec 13;20(12):e1011496. doi: 10.1371/journal.pgen.1011496 (PMC11642917; doi:10.1371/journal.pgen.1011496)
Supplement: S7 Table — (DOCX) [file pgen.1011496.s014.docx]

**Table S7: CRISPR Targeting Sequences and Repair Templates**

| **Gene** | **crRNA** | **crRNA Target Sequence** | **Repair Template** |
| --- | --- | --- | --- |
| *unc-112* | UNC-112::GFP | CTTTTCCACAAACTTACAGG | GCAAAGAACATTCTCAAAATCTTGATGAAGAACTTTTCCATAAGCTCACTGG***AGG***ATGG GCTGGAGGAGGAGGATCCGGAGGAGGAGGATCCGGAGGAGGAGGATCC --- GFP ---  TAGATATTTAAATTTCTATAATCTTTTGCAAACCA |
| *pat-3* | PAT-3::GFP | AAGGATAAAAACTATTTAGT | Hybrid repair strategy - no gene homology repair template  GGAGGAGGAGGATCTGGTGGTGGAGGATCTGGTGGAGGTGGATCA ---GFP----  Long homology repair template  aagtgacacggaacaaaaaacatataaatttatcaaattatcatttttcagAACGAGAACCCAATCTACAAACAGGCCACGACAACATTTAAAAATCCAGTATACGCTGGAAAAG***CCA***ACGGAGGAGGAGGATCTGGTGGTGGAGGATCTGGTGGAGGTGGATCA ---GFP----atagtttttatccttatattttaataattttcccaaattttctaatatgaaagctcaatttctccatccaaacaactcgaaacgagtatttagcgataaattgtcacattcttctttgttttattcaaaaaatct |
| *rpm-1* | RPM-1::mScarlet | GCAGGAGCATTAGTACACGA | Hybrid repair strategy - no gene homology repair template  TCATGCACAAATGCACCTGCAGCTGCAGCGCTTAAGTTGGGAATTCAAGTACCGGTAGAAAAA ---mscarlet---AGCCCACAAGCTTTCACGCGTCCTGGTACCGCTGCAG  Long homology repair template  GGCAGCAGCAGCATATTTGAGATTTCATCAAAACCTTCATAATgtgagtaaagtaaacgttgtttccttacaattaaataaaaaaataatttcagATCTGGCAGTCCTGTGAAATT***CCG***TCATGCACAAATGCACCTGCAGCTGCAGCGCTTAAGTTGGGAATTCAAGTACCGGTAGAAAAA ---mscarlet--- AGCCCACAAGCTTTCACGCGTCCTGGTACCGCTGCAGCTCTCCAACCAATAGTGAAAATTTGGAGAGAAATATGTGAAGTCGTGGAAACCAGTGTAGAGCAGCATTTGATAATGCCCCCAGTTTCAAACAAAGCCATGCGTGCAGAAACTGTGAAGCC |

Legend: underline (crRNA targeting sequence), Italic bold (Pam sequence), Red (Insertion), Blue (silent mutations in repair to prevent Cas9 re-cutting), Orange (linker)
