# Supplementary material for: Integrin adhesome axis inhibits the RPM-1 ubiquitin ligase signaling hub to regulate growth cone and axon development
Source: PLoS Genet. 2024 Dec 13;20(12):e1011496. doi: 10.1371/journal.pgen.1011496 (PMC11642917; doi:10.1371/journal.pgen.1011496)
Supplement: Attachment — Submitted filename: Amezquita and Desbois, response to reviewers.docx [file pgen.1011496.s025.docx]

We appreciate the editor and all three reviewers supportive and valuable comments.

The editor has provided particularly helpful guidance on two major points that were requested for this revised paper that was transferred from *PLoS Biology* to *PLoS Genetics*. 1) They requested that we focus our revised manuscript around interpretation of our genetic and developmental growth cone results. As noted by a reviewer and the editor, our findings indicate that the adhesome components tested, PAT-3, UNC-112 and TLN-1, are inhibiting RPM-1. We agree with this interpretation which substantially focuses the mechanistic conclusions of our study. 2) To further bolster mechanism, we now add new experiments showing that both TLN-1 and RPM-1 effects on axon termination are influenced by pharmacologically altering microtubule stability (**new Fig 8**). In combination with our genetic and developmental time-course studies, these added pharmacological experiments independently support our model that the PAT-3/UNC-112/TLN-1 adhesome axis inhibits RPM-1 to influence microtubule stability. 3) The editor stressed that independent experimental validation (either imaging or coIP results) were important to verify the physical association between adhesome components and RPM-1. We now provide new super-resolution imaging data with CRISPR engineered strains showing that endogenous TLN-1 and RPM-1 co-localize in the axons of *C. elegans* mechanosensory neurons (**new Fig 4, new Supplementary Fig 7**). Please note that this was a major experimental effort for our lab, as we have not previously used super-resolution imaging of any type in a prior study.

Below we provide responses to specific reviewer comments, which also includes commentary on other new experimental results we provide, new data analysis and textual revisions.

**Reviewer #1**

Major comments:

1. Given the well-established role of RPM-1 as an E3 ubiquitin ligase, it is somewhat surprising that there is a lack of mechanistic exploration of RPM-1/integrin network interaction. The fact that the integrin adhesome components are unlikely substrates of RPM-1 suggests that RPM-1 has E3 ligase-independent function, which is an interesting speculation worth of further investigation. For example, what are the protein domains of RPM-1, TLN-1 or UNC-112 required for their physical interaction? Are the growth cone phenotypes in the integrin network mutants associated with defects in microtubule or actin dynamics? What is the role of RPM-1 in these aspects? These experiments will substantially improve the novelty and depth of the paper.

>>>

We appreciate the reviewer noting these important points.

We agree that RPM-1 has a well-established role as a large, atypical RING E3 ubiquitin ligase with key roles in axon termination and synapse maintenance. Our prior work, some of which we cite, has demonstrated that RPM-1 functions as both a signaling hub and ubiquitin ligase. Our findings that RPM-1 functions via ligase independent mechanisms to regulate ANC-1 (Tulgren et al, 2014 PLoS Genetics), RAE-1 (Grill et al, Journal of Neuroscience 2012), GLO-4 (Grill et al, Neuron 2007), and PPM-2 (Baker et al, PLoS Genetics 2014) provide extensive experimental precedent for RPM-1 functioning to regulate downstream signaling via interactions with binding proteins that are not likely to be ubiquitination substrates. Thus, there is much precedent for RPM-1 acting through ubiquitin ligase independent roles to regulate axon termination. As such, we do not go into this further experimentally. However, it is clear from prior studies that little is known about how RPM-1, or any of its orthologs, are regulated. Thus, based on the editor’s comments, this reviewer remarks below, and another reviewer’s interpretation of our genetic results, we now focus our manuscript around the major mechanistic concept that a PAT-3/UNC-112/TLN-1 adhesome axis inhibits RPM-1. As a result, our manuscript represents a substantial step forward for the field. We comment on these specifics of this further below.

With regard to effects on microtubules. We agree that RPM-1 is known to destabilize microtubules, which affects growth cone collapse thereby influencing axon termination. Indeed, these are concepts that our lab previously established using both pharmacological approaches with colchicine treatment (microtubule destabilizing drug) of *rpm-1* mutants which suppresses failed axon termination defects, and genetic studies between *rpm-1* and other well-established destabilizers and stabilizers of microtubule dynamics (Borgen et al, Development 2017). We now include new pharmacological experiments with colchicine showing that RPM-1 and TLN-1 affect axon termination via impacts on microtubule stability (**new Fig 8**). These new results, complement our mechanistic developmental time-course studies on growth cone dynamics in *rpm-1* and *tln-1* mutants (reannotated as **Fig 7**). Thus, our revised paper has increased examination of molecular, developmental and cellular mechanisms. As a result, we draw a more mechanistically driven conclusion that the PAT-3/UNC-112/TLN-1 adhesome axis inhibits RPM-1 to influence microtubule stability, growth cone collapse and axon termination (**new Fig 9**).

Regarding mapping the physical interaction between RPM-1 and the adhesome. This is a reasonable point, but it is difficult to know where to start in addressing this question. We do not assume that adhesome hits in our AP-proteomics with RPM-1 represent direct binding. Hence, our us of the term “physical association”. From our proteomics data it is difficult to know which adhesome component(s) directly bind RPM-1. Trying to address this is beyond the scope of this study. Moreover, without this information attempting to map how interactions occur is also not necessarily informative if we are not dealing with a clear direct binding protein for RPM-1. To address the reviewer’s concern, we turn to an alternative. We now provide super-resolution imaging data to show that endogenous RPM-1 and TLN-1 colocalize in the mechanosensory neurons of *C. elegans* (**new Fig 4; new Supplementary Fig 7**). These new super-resolution imaging results with endogenous CRISPR engineered proteins is key orthogonal/independent validation our proteomic findings. Given the time consuming and costly nature of these new imaging experiments, we considered it beyond the scope of this study to examine whether PAT-3 and UNC-112 also colocalize with RPM-1. However, given similar axonal localization for these adhesome components (**Fig 3**), we think it is likely they would also co-localize if tested.

2. It seems that GS::RPM-1 and GS::RPM-1(LD) used in the paper are integrated multi-copy gene arrays. If so, GS::RPM-1 and GS::RPM-1(LD) are likely to be expressed at high, non-physiological level. Therefore, the authors need to verify physical interaction between RPM-1 and PAT-3, UNC-112 or TLN-1 using a more standard co-immunoprecipitation experiment, with low-copy or single-copy RMP-1 transgene and the endogenously-tagged GFP alleles of pat-3, unc-112 or tln-1.

>>>

The reviewer is correct that we have used integrated multi-copy arrays to express GS::RPM-1 and GS::RPM-1 LD. However, as published previously and cited (Crawley et al, Nat Communs 2019; Desbois et al, PLoS Genetics 2022; Desbois et al, STAR Protocols 2023), these constructs are expressed on an RPM-1 protein null background (Alabdi et al, Brain 2024). Moreover, the constructs are expressed using a native *rpm-1* promoter. Constructs were previously shown to have prominent rescue of *rpm-1* loss-of-function phenotypes (Crawley et al, Nat Communs 2019; Desbois et al, STAR Protocols 2023). Finally, we note that all genetic, developmental and pharmacological studies in our manuscript now point to the PAT-3/UNC-112/TLN-1 adhesome axis as a regulator of RPM-1 during growth cone and axon development. We strongly value integrated proteomic, cell biological, genetic and pharmacological results pointing towards our conclusions and model. Thus, numerous independent approaches validate and support our proteomic findings.

While we understand the reviewer’s point and caveats associated with our proteomics approach, we respectfully ask the reviewer to consider the possibility that their comment might be too negative regarding physiological relevance. In fact, our system does have high physiological relevance, as demonstrated by numerous prior publications that identified functional RPM-1 binding proteins using this AP-proteomics approach (Crawley et al, Nat Communs 2019; Desbois et al, PLoS Genetics 2022; Tulgren et al, PLoS Genetics 2014; Baker et al, PLoS Genetics 2014; Grill et al, Journal of Neuroscience 2012; Grill et al, Neuron 2007). As far as the field is concerned, our lab’s work stands as the leading example of *C. elegans* neural proteomics performed to date. Please note that *C. elegans* is principally a genetic model organism. Our lab has been pioneering efforts in the proteomic and biochemical realm for this model for the past 15 years. AP-proteomics using endogenous CRISPR engineered proteins is something we are presently pursuing and is likely to be valuable in the future. Indeed, we are already seeing promising results with this type of approach using a different ubiquitin ligase that is not part of this study.

With these considerations noted, we have opted to address the reviewer’s point about independent experimental validation of adhesome component physical association with RPM-1 using a different approach. Based on the editor’s guidance and another reviewer’s input, we have done this using an imaging approach. We now use super-resolution imaging with CRISPR engineered endogenous TLN-1::GFP and RPM-1::mScarlet to demonstrate that RPM-1 and TLN-1 co-localize within the axon of PLM neurons in *C. elegans* (**new Fig 4; new Supplementary Fig 7**). Super-resolution imaging was key for these new studies as increased imaging sensitivity was critical in detecting colocalization of endogenous RPM-1 and TLN-1. These experiments required the Grill lab to work for the first time with this type of super-resolution imaging technology, which is a substantial step forward for our lab’s capabilities. Indeed, we consider this no small feat and hope the reviewer appreciates our new data.

3. The authors should provide protein ontology analysis to show that the association of integrin adhesome with RPM-1 is significant, in comparison to other protein signaling networks. For example, is actin or microtubule signaling network also physically associated with RPM-1? How about the endosomal trafficking network (could serve as a negative control)? This analysis is crucial to establish the specificity of such association.

>>>

The reviewer makes a valuable point. To address this, we turn to a comment from another reviewer that requested gene ontology (GO) term analysis for our RPM-1 AP proteomic dataset. We now provide GO process analysis for RPM-1 AP proteomics and integrate this with adhesome components. This is now presented in a new supplemental Figure (**new Sup Fig 1**). This revealed that several of the top 20 GO process terms contain adhesome components.

4. P3-4: Please elaborate more on the AP-proteomics results using GS::RPM-1 and GS::RPM-1::LD (= "ligase-dead"? Please specify). For example, the implications of RPM-1-binding proteins and RPM-1 substrates in axon termination should be substantiated (the first paragraph of Results). There is little background in the Introduction on how RPM-1/PHR/Highwire ubiquitin ligase regulates axon termination by targeting specific substrates for proteasome degradation, and this part definitely needs substantiation.

>>>

AP-proteomics for RPM-1 was previously published and described (Crawley et al Nat Communs 2019; Desbois et al, PLoS Genetics 2022; Desbois et al, STAR Protocols 2023). However, we agree that more attention to experimental detail could be valuable for readers. Thus, we have expanded the text and citations in the first section of the Results. We also point the reviewer to the Methods which has a more lengthy description of AP-proteomics for RPM-1.

Please note that mass spectrometry datasets have also been uploaded onto the PRIDE database, and this is now noted in the Methods:

*“Mass spectrometry files for RPM-1 AP-proteomics (Project number: PXD051783) have been uploaded to the PRIDE database (https://www.ebi.ac.uk/pride/).”*

5. P4: The assumption that "the majority of adhesome components associated with RPM-1 are not likely to be ubiquitination substrates" should be examined by biochemical experiments. The TLN-1::GFP or UNC-112::GFP alleles that the authors created will be perfect reagents to carry out related experiments - likely a western blot experiment showing TLN-1 or UNC-112 bands in the wild type, the rpm-1 mutant and animals that express high levels of rpm-1.

>>>

Our prior studies have shown that GS::RPM-1 LD is an excellent “biochemical trap” that enriches RPM-1 ubiquitination substrates that were previously confirmed genetically and biochemically (Crawley et al, Nat Communs 2019; Desbois et al, PLoS Genetics 2022). Our prior work also indicates that RPM-1 is known to bind and function via proteins such as ANC-1, PPM-2, RAE-1 and GLO-4 that bind RPM-1 but do not behave as ubiquitination substrates (Tulgren et al, PLoS Genetics 2014; Baker et al, PLoS Genetics 2014; Grill et al, Journal of Neuroscience 2012; Grill et al, Neuron 2007). Thus, we rely upon this extensive prior precedent to argue that adhesome components are likely to bind but not be ubiquitination substrates, a point we make with Table 1.

As noted by the editor and another reviewer, our genetic results indicate that the PAT-3/UNC-112/TLN-1 adhesome axis functions as an upstream inhibitor of RPM-1. We agree with these points and have reshaped our paper around this important concept. However, these genetic findings argue strongly against the possibility that adhesome components are RPM-1 substrates.

We agree that it would be beneficial to provide further biochemical experiments. However, in pursuing these for many months using CRISPR engineered and transgenic proteins, we have not generated sufficiently consistent results for presentation in this paper. This potentially reflects that we are not sure about the adhesome components that are direct binding proteins for RPM-1. We hope to address this with future biochemical studies in more detail. Indeed, this is the first evidence that the adhesome interacts with and regulates RPM-1. As the reviewer notes, there is much valuable work possible which we are planning to pursue in the future.

6. P7: The genetic experiments of tln-1 in Figure 4 could be further substantiated. (1) Since the authors indicate that ok1648 is a hypomorphic allele (Figure 4D), this could be an in-frame or small/partial deletion of the tln-1 gene. Although a diagram is provided in Figure S4, some more explanation is helpful, such as whether ok1648 causes a frame shift for isoform a, the length of the deletion, etc. (2) Although it is explained in the method section, please also indicate in the Results section that tln-1 rescue is achieved using a genomic tln-1 fragment. (3) The authors should try to rescue with only tln-1a or tln-1b to test whether either isoform alone is sufficient to rescue, or isoforms a and b have their own unique functions that are not exchangeable.

>>>

We appreciate the reviewer’s point. We have now performed our own sequencing of the ok1648 allele, which confirms information noted on Wormbase (https://wormbase.org). Our results indicate that ok1648 results in a deletion that affects TLN-1 isoform a, but does not affect the coding sequence of TLN-1 isoform b. This new point about independent verification for the ok1648 allele is included as new text of the Methods as follows:

*“We sequenced the tln-1(ok1648) allele and verified that is affects TLN-1 isoform a, c, d and e but does not affect the coding sequence for TLN-1 isoform b and f. This independently confirms what is annotated on WormBase (https://wormbase.org).”*

These results confirm it is reasonable to consider the *ok1648* allele a hypomorph, as it is unlikely to affect TLN-1 isoform B. Our claim that *ok1648* is a hypomorph is further supported by the higher frequency defects that are observed with *mecDEG* targeting of TLN-1 compared to results with *tln-1 (ok1648)* (**Fig 5D**)*.*

While we appreciate the suggestion of performing transgenic rescue experiments with TLN-1a and b isoforms, we have opted to focus on the extensive number of other experiments requested by this reviewer and others that more heavily impact the mechanism and conclusions of our study. We hope to further address the role of different TLN-1 isoforms in axon termination as part of a future study.

To further address this point, we have softened our language in the Results by adjusting the following statement:

*“Thus, our findings suggest that TLN-1a and b isoforms are particularly important for axon termination. Moreover, our observation that tln-1(ok1648) mutants have lower frequency premature termination defects is likely to reflect that this allele is hypomorphic.”*

7. P9: Authors' interpretation of the genetic interaction between rpm-1 and the integrin adhesome genes (pat-3, unc-112, tln-1) needs further clarification and substantiation. (1) The PLM growth cones in most of the rpm-1; TLN-1::mecDEG animals failed to collapse during larval development, similar to those in the rpm-1 mutant. This suggests that, for proper growth cone collapse, tln-1 acts upstream and antagonizes rpm-1. (2) Same as (1): the axon termination phenotypes are also consistent with that the adhesome components limit RPM-1 activity and act upstream of rpm-1 downstream. (3) It is intriguing that the premature axon termination phenotype of the rpm-1; TLN-1::mecDEG animals gradually shifts to the failed termination phenotype, from 16 h to 44 h post-hatching. The authors explained this from the viewpoint of a tug-of-war model, but this does not explain how the phenotype evolves from one extreme to the opposite extreme over time. This part should be further substantiated. One caveat of the ZIF-1-based degron in the current study is the lack of temporal control of protein degradation. The auxin-inducible degradation (AID) should allow them to test the role of the integrin adhesome components at specific developmental time points.

>>>

We appreciate the reviewer making this important point. We agree entirely that our genetic results and developmental time course studies on growth cone dynamics support the conclusion that TLN-1, and the PAT-3/UNC-112/TLN-1 adhesome axis, function as an upstream inhibitor of RPM-1. In fact, based on this feedback and similar comments from the editor and another reviewer, we have opted to substantially rewrite our manuscript and revise the title of our paper. Based on new data on TLN-1 and RPM-1 effects on microtubule stability during axon termination (**new** **Fig 8**), existing developmental time course studies on growth cone collapse (**Fig 7**), and new super-resolution imaging showing co-localization of TLN-1 and RPM-1 in axons (**new Fig 4; new Supplementary Fig 7**), our study now generates a more mechanistically driven primary conclusion that the PAT-3/UNC-112/TLN-1 adhesome axis inhibits RPM-1 to influence microtubule stability, growth cone collapse and axon termination. This is now reflected in a new summary diagram of our findings (**new Fig 9**), major revisions to the text of our manuscript, and an improved and more mechanistically driven title for our study.

We acknowledge the reviewer’s valid point that the ZIF-based degron we use in our study does not give us temporal control over degradation of adhesome components. However, we consider employing an auxin-inducible degradation system to be outside of the scope of this manuscript. With extensive, major new revisions and new experimental data, we hope the reviewer agrees that our study is now substantially more mechanistic and conclusive.

Minor comments:

1. P4: Please provide justification for the criteria of defining integrin adhesome proteins in C. elegans, such as 30% protein sequence similarity, 20% sequence identity, etc.

>>>

We appreciate the reviewer’s comment and will begin by noting what was done previously in a prior bioinformatic paper by Zaidel-Bar R. (*Journal of Cell Biology*, 2009). Zaidel-Bar used the following criteria for identifying potential *C. elegans* orthologs for human adhesome components:

1) *C. elegans* ortholog has sequence similarity along at least 50% of the protein (bit score above 80).
2) Ortholog contains all major protein domains, as detected by the conserved domain database (Marchler-Bauer et al., 2007).
3) *C. elegans* ortholog is the top reverse BLAST against the human genome.
4) Certain *C. elegans* proteins were considered orthologs based on the literature, even though they did not satisfy all the above criteria.

Our updates to the *C. elegans* adhesome were based on the most up to date computational databases (Alliance of Genome Resources, WormBase) and careful re-examination of prior annotation assigned by Zaidel-Bar (Zaidel-Bar R. et al, *Nature Cell Biology* 2007). We have now updated the Results and Methods to note our use of Alliance of Genome Resources and WormBase as resources we used to revisit the *C. elegans* adhesome.

Using the prior 232 components that comprise the human adhesome network (Zaidel-Bar R. et al, *Nature Cell Biology* 2007), we independently identified *C. elegans* adhesome orthologs based on four primary criteria:

1) *C. elegans* ortholog having 30% or higher sequence similarity

2) *C. elegans* ortholog having 20% or higher sequence identity

3) Prior annotation in the *C. elegans* adhesome, which we carefully re-evaluated here ourselves. (Zaidel-Bar R, *Journal of Cell Biology* 2009).

4) Using the Alliance of Genome Resources to independently confirm the *C. elegans* ortholog as the top reverse BLAST hit against the most recent annotation of the human genome.

2. Figure 1B: The 1.5-fold dashed line is not intuitive. If the standard 1-fold (no change) orthogonal line is used, one could easily see that data points biased towards the GS::RPM-1 (v.s. GS::GFP) as being enriched.

>>>

The 1.5 fold dashed line represents proteomic hits that are enriched 1.5x in test GS::RPM-1 samples compared negative control GS::GFP samples (**Fig 1B**). We prefer a more stringent cutoff than the standard 1x fold enrichment, which increases the rigor of our study. In our prior figure, data presentation for hits at similar spectral thresholds were distributed such that some hits appeared less than 1.5x enrichment when this is not the case. We have updated our data point distribution accordingly to reflect this. We appreciate the reviewer bringing this to our attention.

To further address this point, we now include Supplemental Data File 1 which includes all our unnormalized and normalized spectral counts for all hits in RPM-1 AP-proteomics experiments. We also now include Supplemental Data File 2, which shows all statistical analysis for normalized mass spectrometry data.

3. In Figure 3D-G, the localization of PAT-3, UNC-112 and TLN-1 in the mechanosensory neurons seems to be diffuse. How is this distribution pattern compared to that of RPM-1? Can the authors explain why these integrin adhesome components show such diffuse distribution patterns?

>>>

We appreciate the reviewer’s point. Perhaps they were wondering why we do not see puncta representing focal adhesions where adhesome components would be enriched. This could simply reflect the mechanosensory neurons of *C. elegans* (and *C. elegans* neurons in general) which are very small compared to mammalian neurons. For example, for markers of microtubules and actin it is very hard to image structures such as F-actin enrichment/focal adhesions or the dynamic ends of microtubules in *C. elegans* mechanosensory neurons (Borgen et al, *Development* 2017). In contrast, these structures are relatively easily observed in the much larger growth cones and axons of cultured mammalian neurons.

To further address this comment, we now provide super-resolution Zeiss Airyscan imaging for CRISPR engineered endogenous GFP::TLN-1 and RPM-1::mScarlet (**new Fig 4; new Supplementary Fig 7**). Our results yield two key points:

1) We now show that RPM-1 is localized and concentrated at the termination tip of mechanosensory neurons where we detect colocalization with GFP::TLN-1. Thus, we can observe RPM-1 and TLN-1 in similar axonal compartments in mechanosensory neurons, which is consistent with our proteomic and genetic findings.

2) Even with greater resolution, GFP::TLN-1 CRISPR is still diffusely localized in the mechanosensory axons. While even higher resolution technologies might reveal TLN-1 puncta, we do not have access to this technology, and we are would not necessarily see focal adhesions along the axons. Again, this could be due to the small size of *C. elegans* neurons. One alternative is that diffuse adhesome component localization could be occurring because mechanosensory neurons are embedded between the muscle and epidermal cell yielding extremely high frequency adhesion sites. A second alternative might be that the locations of GFP tags or GFP’s size might not be impairing function but could be reducing precision of localization. While we appreciate these subtleties, we do not think they change our conclusions in any way.

4. P6: The definition of premature PLM axon termination is misleading, as "...PLM axon terminated growth at or prior to the vulva..." seems to also imply axon termination anterior to the vulva. Please consider changing it to "....terminated at or posterior to the vulva".

>>>

We appreciate the reviewer making this helpful comment. We have now adjusted the language at three points in the revised paper to the following:

*“PLM axons terminated growth at or posterior to the vulva”*

**Reviewer #2:**

1.      Is the manuscript about the RPM-1 ubiquitin ligase or about the integrin adhesome? The title indicates the latter, but the organisation of the manuscript suggests the former. The rationale to link the two aspects has not been adequately developed in the introduction section. From the literature it wouldn't be hard to make a case to study integrin adhesome components in axon development in C .elegans. Therefore, what is the relevance of RPM-1?

>>>

The reviewer’s point about the focus and impact of our studies is reasonable and we agree that our initial manuscript was a touch lacking in focus.

To address this and to respond to the editor and another reviewer’s comments, we have substantially revised the writing of our manuscript. We now emphasize a more mechanistically driven conclusion that the PAT-3/UNC-112/TLN-1 adhesome axis inhibits RPM-1 to influence microtubule stability, growth cone collapse and axon termination (**new Fig 9**). Thus, abstract, title and discussion now flow more clearly around this concept and place greater emphasis on the substantial step forward our paper represents for the field of adhesome biology.

2.      The study is based upon data regarding RPM-1 interactors / substrates that was generated and published previously. The authors display this data to highlight integrin adhesome components in Figure 1. My feeling is that this has left the manuscript imbalanced in its flow and overall message and I don't think your title accurately reflects the study or evidence obtained from the data shown.

>>>

We appreciate the reviewer’s point. However, we respectfully disagree. The lab has previously published 2 studies using proteomics to identify UNC-51 and CDK-5 as RPM-1 substrates. Here, we pivot to more extensive data analysis with adhesome components. We consider the proteomics and mass spectrometry a key body of data for this study. We have cited prior papers where a small portion of our AP-proteomics data with RPM-1 was published. Please note that in this study we present a new example of an individual proteomics experiment that was not published previously. With 7 independent proteomics experiments, we are able to present cumulative data for individual proteomic hits while presenting primary data for individual proteomics experiments for the first time. We must stress that the proteomics data presented as dot plots in Figure 1B and 1C have not been published before. This is a key advantage and important point about the large-scale nature of our proteomics data. We hope the reviewer can appreciate this.

To further address this comment, we have deposited our mass spectrometry files for our entire RPM-1 AP-proteomics dataset to the publicly accessible PRIDE mass spectrometry database:

*“Mass spectrometry files for RPM-1 AP-proteomics (Project number: PXD051783) have been uploaded to the PRIDE database (https://www.ebi.ac.uk/pride/).”*

3.      The authors do not attempt to validate the interactions of adhesome components with RMP-1 by orthogonal methods. Have the authors attempted col-localisation or co-immunoprecipitations of RPM-1 with any of the identified components? This could be performed in simpler cell biological models. Further discussion should be directed to the relevance of proteins interacting with RPM-1 that are not ubiquitin ligase substrates.

>>>

We appreciate the reviewer’s valuable suggestion. Another reviewer and the editor have also asked us to provide orthogonal experimental validation for physical associations between adhesome components and RPM-1 identified by *C. elegans* proteomics.

1) We appreciate the reviewer’s suggestion of using imaging as an alternative approach for orthogonal experimental validation of proteomics. We now include new super-resolution imaging data using Zeiss Airy-scan technology (**new Fig 4; new Supplementary Fig 7**). Super-resolution imaging with CRISPR engineering players showed that endogenous GFP::TLN-1 colocalizes with RPM-1::mScarlet in the axons of *C. elegans* mechanosensory neurons. Please note that this was the first time that the Grill lab has used super-resolution imaging, and the first time we have generated and worked with CRISPR engineered RPM-1::mScarlet. Thus, this experimental version was a very heavy lift for our team. Nonetheless, we think the results are extremely valuable, substantially strengthen our study and have made the extensive effort involved worthwhile.

2) Please note that we very carefully chose the term “physical association” when discussing our proteomic data rather than “RPM-1 binding protein” (language we have used in past papers). We think “physical association” accurately captures the absence of further biochemical experiments in our study without diminishing findings from an unusually large, valuable proteomics dataset. While the reviewer might not be aware, proteomics is rarely used in the *C. elegans* model in particular for studies in the nervous system. Thus, for our model, the proteomics shown is not only valuable but stretching our model to its maximum potential.

3) We are dealing with numerous adhesome components physically associated with RPM-1. Which adhesome components are potential direct binding protein(s) for RPM-1 is not clear from our proteomics results. Thus, we have opted against using biochemistry to further test physical associations. In our hands, indirect protein interactions can be very hit or miss on sensitivity for in vivo biochemistry from *C. elegans*. Thus, without a better sense from proteomics about which adhesome component might be a direct binding protein, we have not performed what would be an extremely long, difficult series of experiments.

We note this caveat about our proteomic data with the following new text in our Results section:

*“While the specific adhesome components that directly bind RPM-1 remain unclear, we anticipate this would only occur for a few components with indirect physical associations occurring between RPM-1 and the majority of adhesome players detected.”*

4.      Methods to describe the AP proteomics strategy are lacking. The authors do helpfully direct the readers to previous published manuscripts for this information but the necessary details to understand the findings made in this paper are missing. Has the dataset been deposited in a proteomics repository? This is usually required as a condition for publication. Please provide details.

>>>

We appreciate this important point. We have now expanded the Results to make this clearer with the following updated text:

*“To identify proteins physically associated with the RPM-1 ubiquitin ligase signaling hub, we previously performed large-scale, unbiased AP-proteomics using C. elegans [26, 34]. We relied upon integrated transgenes that express RPM-1 fused with a Protein G::Streptavidin binding peptide (GS) affinity tag expressed using the native rpm-1 promoter on an rpm-1 protein null background. Negative control animals expressed an integrated transgene where the GS tag was fused to GFP (GS::GFP) and expressed by the rpm-1 promoter. AP-proteomics was performed with two RPM-1 constructs: GS::RPM-1 and GS::RPM-1 ligase-dead (LD). RPM-1 LD is point mutated in its RING ubiquitin ligase domain, which inactivates catalytic ligase activity and enriches ubiquitination substrates [26, 35]. We previously established two groups of RPM-1 associated proteins identified by AP-proteomics, RPM-1 binding proteins and RPM-1 ubiquitination substrates (****Fig 1A****) [26, 35]. RPM-1 binding proteins (e.g. RAE-1, GLO-4, FSN-1 and PPM-2) are present in both the GS::RPM-1 and GS::RPM-1 LD samples compared to the GS::GFP negative control* ***(Fig 1B****) [26, 35-38]. In contrast, RPM-1 LD biochemically ‘traps’ and enriches ubiquitination substrates (e.g. ULK/UNC-51 and CDK-5) (****Fig 1C****) [26, 35].”*

Please note that all RPM-1 AP-proteomics mass spectrometry files have been uploaded to the PRIDE database to make this data publicly accessible. This is now noted in the revised Methods with the following text:

*“Mass spectrometry files for RPM-1 AP-proteomics (Project number: PXD051783) have been uploaded to the PRIDE database (https://www.ebi.ac.uk/pride/).”*

5.      The choice of detergent used for lysis before the RPM-1 AP and proteomics is critical. This reviewer finds it very difficult to believe that RPM-1 interacts directly with all the adhesome components identified. In fact it is very difficult to purify adhesomes as they are labile structures. Which is the critical link from RPM-1 to the adhesome? Why do the authors think all the adhesome components co-purify with RPM-1? Further discussion is needed.

>>>

To be clear, we do not see “all” adhesome components in RPM-1 AP-proteomics. We see a subnetwork of adhesome components (**Fig 2B**, note adhesome components shown as grey circles were not detected in RPM-1 AP proteomics).

We agree with the reviewer that the physical associations between multiple adhesome components and RPM-1 are likely to be indirect. We now address this with new text in the Results that makes it very clear that we agree with this point:

*“While the specific adhesome components that directly bind RPM-1 remain unclear, we anticipate this would only occur for a few components with indirect physical associations occurring between RPM-1 and the majority of adhesome players detected.”*

Thus in our revised manuscript, it is now clearer that we have identified multiple adhesome components because a subnetwork of the adhesome is indirectly and directly interacting with RPM-1. Indeed, our prior studies indicate that RPM-1 is a signaling hub with many binding proteins and ubiquitination substrates. This is evident in the dot plot in Figure 1B showing data for 1 of 7 proteomics experiments, and prior publications from our lab and others (e.g. Tulgren et al, PLoS Genetics 2014; Baker et al, PLoS Genetics 2014; Crawley et al, Nature Communs 2019; Desbois et al, PLoS Genetics 2022; Grill et al, Journal of Neuroscience 2012; Grill et al, Neuron 2007).

We appreciate the reviewer’s point about the adhesome being labile and hard to purify. This is consistent with our findings. In a prior protocol paper for *C. elegans* proteomics (Desbois et al, *STAR Protocols* 2023), we make the point that rapid affinity purification (1-4 hours), use of varying detergent conditions, and the use of a cryomill to generate worm grindates under liquid N2 temperatures is critical for successful *C. elegans* neural proteomics.

The reason we have such large-scale proteomics data sets with seven independent RPM-1 AP-proteomics experiments is because we have used a range of detergents and detergent concentrations for our experiments. Our data is derived from two experiments with *C. elegans* extracts made using 0.1% NP-40, 0.3% NP-40 or 0.1% CHAPS. Consistent with the reviewer’s keen instincts, we have used extremely rapid extraction under liquid N2 temperature, and used relatively low and variable detergent conditions. We now note the buffer and detergent concentrations for individual proteomics experiments in **new Supplemental Data File 1**, along with the prior protocol paper where this is covered in extensive detail. We hope these points of clarification prove valuable for the reviewer and the field in determining if particular detergent conditions might be helpful for extracting and examining different types of adhesome components.

6.      Figure 1. I would suggest the mass spectra to confirm the identification of the three adhesome proteins are not necessary. They could be moved to a supplementary figure if required. More important would be to use this figure to show the spectral counts (quantitation) for each AP experiment / adhesome protein to demonstrate the confidence and variability in this measure.

>>>

While we understand the reviewer’s desire to remove mass spectra, we consider this raw data to be valuable. Displaying the mass spectra allows readers to quickly understand we are dealing with proteomics. It also is a presentation of the actual raw data that underpins our key adhesome players that we study further in subsequent experiments.

To further address this comment, we have now included new Supplementary Data Files 1 and 2 that comprehensively list adhesome components identified, spectral counts for individual experiments and provide our statistical analysis.

7.      The authors use two different approaches to identify enriched adhesome components in their AP dataset. They switch between using a 1.5 fold spectral count cut off and a statistical p-value cut-off. Please select one or justify the switch. This is important in Figure 2 where the authors claim that 75% of the identified adhesome components have genetic links to human neurobehavioral abnormalities. This depends on how the selection / cut-off was made. Also is the statistical test used appropriate? and what happens with missing values or multiple test corrections? Would a non-parametric test be more appropriate?

>>>

We appreciate the reviewer’s comment which is valuable and allowed us to significantly improve the quality of our analysis and presentation of proteomics data with the following updates.

1) We now include language in the Figure 1 legend to specify that the dashed grey line in Figure 1B is the 1.5x fold enrichment based on total spectra without normalization.

“*Grey dashed line represents 1.5x enrichment of spectra for proteomic hits in GS::RPM-1 sample compared to GS::GFP negative control.”*

This is based on an initial threshold for inclusion. All further data analysis and statistical testing is done using normalized proteomics data as specified in the Methods and Legends. We consider it more rigorous to rely upon statistical analysis with normalized data rather than fold enrichment for GS::RPM-1 versus GS::GFP (negative control). We keep fold enrichment as a metric with normalized data for GS::RPM-1 versus GS::RPM-1 LD, as we previously showed this is a valuable metric for assessing if a proteomic hit could be a putative substrate.

2) We have re-analyzed our data with a non-parametric statistical test, Mann-Whitney, as requested on normalized proteomics data. This is now incorporated into our data presented in Figure 2, Table 1 and Supplemental Data Files 1 and 2. We also note this where appropriate in our figure legends.

3) We have updated Table 2 with several changes to data analysis and presentation. Our column

for “Significance GS::RPM-1 versus GFP::GFP (p-value)” now has p value data generated using a Mann-Whitney non-parametric test rather than a parametric Student’s t test.

We include a new column for “Significance GS::RPM-1 versus GS::GFP (q-value)”. Here we have tested our normalized proteomics data with Mann-Whitney and *post hoc* Benjamini-Hochberg method (*q*-value) with a 5% false discovery rate (FDR) (see Table 1 legend and Methods).

4) We have updated data and significance values in Figure 2B based on our new statistical analysis. Here, we did not adjust for multiple comparisons as this is covered in the Table 1 and we want to ensure the field clearly has certain potentially stronger hits flagged in this diagram.

5) We only examined if positive hits in RPM-1 AP proteomics were encoded by genes associated with neurobehavioral abnormalities. As such, we concluded that 75% of the adhesome components physically associated (proteomic hits) with RPM-1 are linked to neurobehavioral disorders. We consider this important to ensure the field can clearly see several adhesome components detected in RPM-1 AP proteomics and RPM-1/MYCBP2 have links to human neurobehavioral abnormalities. Our goal here is not to make a compelling quantitative point about links to neurobehavioral abnormalities for this subset of adhesome players. Rather, we simply wish to bring this to the attention of the field so they are aware of these potentially important clinical implications.

8.      Figure 2. The authors show a network created from a bioinformatic generation of a C. elegans adhesome. This is a valuable resource and is underplayed in the manuscript. It would also be helpful to provide this information in a format that can be easily used by others e.g. not a pdf table. Other supplementary data tables should also be reformatted so they are not pdf tables. Perhaps the authors could contact the adhesome.org authors to add this data to their website?

>>>

We appreciate this important comment. We now include our Supplementary Tables as Excel and Word files to ease in data analysis and uploading by other groups.

Based on this valuable comment, we have also noted our independent curation and update for the *C. elegans* adhesome.

9.      On page 4 the authors state "Our results highlight the integrin receptor cluster formed by b-integrin PAT-3/ITGB1, a-integrin PAT-2/ITGA8, the Kindlin complex cluster (UNC-112/FERMT1, PAT-4/ILK, UNC-97/LIMS, PAT-6/PARVA), and the Talin complex cluster (TLN-1/TLN1, DEB-1/VCL) (Fig 2A, B)." Here the authors claim that the mammalian orthologue of PAT-2 is ITGB8. Is this correct? Please check as the manuscript from Brown NH (Matrix Biology Volume 19, Issue 3, 1 July 2000, Pages 191-201) states that this integrin could be other RGD binding integrin subunits such as integrin aV, integrin aIIb or integrin a5

>>>

We appreciate the reviewer making this careful observation and valuable comment. This is a somewhat tricky point as the Alliance of Genome Resources and WormBase databases note that there are 4 mammalian alpha integrin proteins (ITGA5, ITGAV, ITGA8 and ITGA2b) that are all likely to be orthologs of an ancient single *C. elegans* PAT-2 protein. ITGA5, ITGAV and ITGA8 are mammalian adhesome components. Please note that to our knowledge ITGA2 is in the mammalian adhesome but not ITGA2b.

To address this point in our revised manuscript, we used the terminology “ITGA” in Table 1 and Figure 2b. We also add the following new text to our manuscript: “*We note that C. elegans PAT-2 has three very similar mammalian orthologs in the adhesome: ITGA5, ITGAV and ITGA8 (****Table S1****)*. Finally, in our revised Supplemental Table 1, we now note that PAT-2 is orthologous to the ITGA5, ITGAV and ITGA8 mammalian adhesome components.

10.     In this reviewer's opinion the in vivo genetic intervention / imaging experiments (figures 3-6) do not convincingly demonstrate that the adhesome components and RPM-1 functionally interact. They show that both are involved in biological processes that regulate axon function but not that RMP-1 and the adhesome function together in any way. Therefore the term 'tug-of-war' should be rephrased.

>>>

We understand the reviewer concern.

However, another reviewer, the editor and our group consider our genetic results and developmental time course studies on growth cone dynamics to support the conclusion that TLN-1, and the PAT-3/UNC-112/TLN-1 adhesome axis, function as an upstream inhibitor of RPM-1. As a result, we have opted to substantially rewrite our manuscript and revise the title of our paper. Based on new data on TLN-1 and RPM-1 effects on microtubule stability during axon termination (**new** **Fig 8**), existing developmental time course studies on growth cone collapse (**Fig 7**), and new super-resolution imaging showing co-localization of TLN-1 and RPM-1 in axons (**new Fig 4; new Supplementary Fig 7**), our study now generates a more mechanistically driven primary conclusion that the PAT-3/UNC-112/TLN-1 adhesome axis inhibits RPM-1 to influence microtubule stability, growth cone collapse and axon termination. This is now reflected in a new summary diagram of our findings (**new Fig 9**), major revisions to the text of our manuscript, and an improved and more mechanistically driven title for our study.

We hope our new more extensive data and clearer presentation will encourage the reviewer to better see this favored interpretation of our results. While we do not disregard the reviewer’s point, we have opted to structure our manuscript to address the majority perspective held by another reviewer, the editor and ourselves.

a.      Figure 3: structures predictions are not required in the figure.

>>>

We think these structural predictions support the point we make around tags not affecting protein folding and viability. As we note in our manuscript, the null mutants for PAT-3, UNC-112 and TLN-1 are not viable. Therefore, we opted to keep these structural predictions to support our claims.

These structural predictions also help the reader understand our tools.

Finally, one of the authors put much time and effort into these structural predictions. Removing this data would reduce this authors contribution and could affect their inclusion in our study. These structures cannot be repurposed for another study.

b.      Figure 4: is there a way to demonstrate the removal of the adhesome proteins using this protein degradation system?

>>>

Given the extensive revisions and large amount of new data that was added to this revised manuscript as well as editorial input on how to prioritize our revisions, we did not pursue this further. However, we would like to note several important points that address this concern. 1) Premature axon termination defects were observed with cell-specific degradation of TLN-1 (GFP::TLN-1 CRISPR + *mecDEG*) and with the TLN-1 hypomorphic allele (*ok1648*) (**Fig 5D**). 2) We were able to rescue premature termination defects caused by degradation of TLN-1 with a TLN-1 transgene (**Fig 5D**). 3) We provide extensive controls for this CRISPR-based degradation system including PAT-3::GFP, UNC-112::GFP and GFP::TLN-1 alone controls and *mecDEG* alone controls (**Fig 5D**). 4) In the double mutant, we can observe the *tln-1* mutant phenotype (premature termination) during larval stages, the RPM-1 phenotype (failed termination) does not become prevalent before adulthood (**Fig. 7**). These observations suggest that the degradation of TLN-1 is efficient in the double mutant. 5) Finally, we provide new data showing that axon termination phenotypes caused by both *rpm-1* (lf) or *mecDEG* targeting of TLN-1::GFP CRISPR are influenced by pharmacological perturbation of microtubule dynamics (**new Fig 8**). Thus, independent pharmacological approaches indicate that both RPM-1 and TLN-1 affect axon termination via differential effects on microtubule stability. This provides further mechanistic insight into why the PAT-3/UNC-112/TLN-1 adhesome axis inhibition of RPM-1 affects axon termination. This is mechanistic relationship is now summarized (**new Fig 9**).

c.      Figure 4: does adhesome protein degradation affect RPM-1 localisation?

>>>

The reviewer’s point is well taken. Our substantially expanded body of data support the conclusion that the PAT-3/UNC-112/TLN-1 adhesome axis inhibits RPM-1. While effects on RPM-1 localization are possible, we suspect that a positive regulator of RPM-1 function would be more likely to regulate RPM-1 subcellular localization than a negative regulatory axis like PAT-3/UNC-112/TLN-1.

We now include new super-resolution imaging data with CRISPR engineered components (**Fig 4; new Supplementary Fig 7**) showing that endogenous RPM-1::mScarlet and GFP::TLN-1 CRISPR co-localization at the tips of mechanosensory axons. At present, it remains unknown how RPM-1 localization to subcellular axonal compartments is regulated in any system. Thus, the reviewer poses an interesting question, but given the substantially mechanistic nature of our paper, we opted to consider pursuing this question in the future. We were also guided by editorial input that encouraged us to focus our revisions on other experimental priorities.

d.      Does removal of RPM-1 affect the localisation of any adhesome components?

>>>

We appreciate the value of the inverse inquiry about localization. Given the relatively broad distribution of TLN-1, PAT-3 and UNC-112::GFP CRISPR distribution in axons, even under super-resolution microscopy, we did not think this experiment was likely to be fruitful. It is possible the levels of PAT-3/UNC-112/TLN-1 adhesome axis components could be altered in *rpm-1* mutants. However, this is not likely for several reasons. Our proteomic and genetic results do not indicate that the PAT-3/UNC-112/TLN-1 axis is likely to be ubiquitinated, degraded or function downstream of RPM-1. In this case, we would have expected suppression of defects caused by *rpm-1* (lf) in double mutants with adhesome components. We did not observe this in any of the multiple phenotypic contexts we examined including axon termination, growth cone collapse or growth cone size. In fact, the opposite was observed with *rpm-1* phenotypes dominating in all cases in double mutants supporting the conclusion that RPM-1 is downstream of the PAT-3/UNC-112/TLN-1 adhesome axis.

e.      Page 7: 'We also observed significant premature termination defects in tln-1 (ok1648) mutants compared to wild-type animals (Fig 4E).' There is no Figure panel 4E.

>>>

We appreciate the reviewer noting this error. It is now corrected and this data is know shown solely in Figure 5D.

11.     Page 6: 'We note that TLN-1 has 7 isoforms' please provide evidence or reference. Which species does this refer to?

>>>

There are 7 potential isoforms of TLN-1 in *C. elegans.*

This is annotated on Wormbase (https://wormbase.org). Below is a screenshot from Wormbase that addresses this point. We also annotate all TLN-1 isoforms as a schematic in Supplemental Figure 5.

**Reviewer #3**

1) Figure 2 clearly shows that PAT-3, UNC-112 and TLN-1 are present along axons of mechanosensory neurons. Is this localization modified in RPM-1 mutants? Conversely, is the axonal localization of RPM-1 modified in the absence of adhesome components?

>>>

The reviewer’s point is well taken.

Based on the extensive revisions requested by all reviewers and editorial input, we have opted to focus on other experiments. However, our revised paper now provides substantially expanded data to further support the conclusion that the PAT-3/UNC-112/TLN-1 adhesome axis inhibits RPM-1. We now include new super-resolution imaging data with CRISPR engineered components (**new Fig 4**) showing that endogenous RPM-1::mScarlet and GFP::TLN-1 CRISPR co-localization at the tips of mechanosensory axons. New pharmacological data demonstrates that both RPM-1 and TLN-1 affect axon termination via effects on microtubule dynamics (**new Fig 8**). Our existing data also shows that all three primary phenotypes caused by *rpm-1* (lf) (failed axon termination, impaired growth cone collapse and increased growth cone size) are present in double mutants for *rpm-1* and *tln-1* (*rpm-1;* GFP::TLN-1 + *mecDEG* - **Fig 6, Fig 7**). In contrast, phenotypes caused by impairing TLN-1 (GFP::TLN-1 + *mecDEG*) are suppressed when *rpm-1* is also impaired.

Given that the PAT-3/UNC-112/TLN-1 adhesome axis inhibits RPM-1 it is possible that RPM-1 localization could be regulated by this adhesome axis. At present, it remains unknown how RPM-1 localization to subcellular axonal compartments is regulated in any system. In this study, we now put forward an entirely new regulatory mechanism, the PAT-3/UNC-112/TLN-1 axis, that inhibits RPM-1. Going beyond this into further subcellular localization studies was beyond the timelines and scope of this paper given the extensive revisions performed, the substantial textual revisions that were needed, and the altered venue where our paper is under consideration.

Regarding effects of RPM-1 on PAT-3/UNC-112/TLN-1 localization. It is also a reasonable question. Given the relatively broad distribution of TLN-1, PAT-3 and UNC-112::GFP CRISPR in axons (even using our new super-resolution microscopy techniques), we did not think it is likely impairing RPM-1 will affect the subcellular localization of components in the PAT-3/UNC-112/TLN-1 axis. It is still possible the levels of PAT-3/UNC-112/TLN-1 adhesome axis components could be altered in *rpm-1* mutants. However, this is not likely for several reasons. Our proteomic and genetic results do not indicate that the PAT-3/UNC-112/TLN-1 axis is likely to be ubiquitinated, degraded or function downstream of RPM-1 (see manuscript for extensive new commentary on this). In this case, we would have expected suppression of defects caused by *rpm-1* (lf) in double mutants with adhesome components. We did not observe this in any of the multiple phenotypic contexts we examined including axon termination, growth cone collapse or growth cone size. In fact, the opposite was observed with *rpm-1* phenotypes dominating in all cases in double mutants, which supports the conclusion that RPM-1 is downstream of the PAT-3/UNC-112/TLN-1 adhesome axis. A conclusion that was favored by another reviewer, the editor and us.

We hope the reviewer can also understand that super-resolution imaging with CRISPR engineered proteins was a major experimental effort for our lab. We have not previously used super-resolution imaging of any type in any prior study. We prioritized this series of experiments based on editorial input and comments from two other reviewers.

2) The authors use an elegant and innovative approach to degrade adhesome components specifically in mechanosensory neurons. While the phenotypes described in Fig. 4 are clear and convincing, they do not indicate how efficient the targeted degradation of each component is. Is GFP completely lacking or can it still be detected at lower levels in the axons of PLM neurons in transgenic knock-in animals? Similarly, it is important to show that the degradation system is still efficient in RPM-1 mutants and effectively leads to a lack of or reduced GFP along axons in double mutants.

>>>

We appreciate the reviewer’s appreciation of the innovative nature of our CRISPR-based cell-specific degradation approach (*mecDEG*) for targeting PAT-3, UNC-112 and TLN-1 specifically in mechanosensory neurons. As the reviewer is aware, this has allowed us to avoid lethality caused by null alleles for PAT-3, UNC-112 and likely TLN-1, and study the role of the PAT-3/UNC-112/TLN-1 adhesome axis in axon termination and growth cone development specifically in mechanosensory neurons.

Given the extensive revisions and large amount of new data that was added to this revised manuscript as well as editorial input on how to prioritize our revisions, we did not pursue this further. However, we would like to note several important points that address this concern. 1) Premature axon termination defects were observed with cell-specific degradation of TLN-1 (GFP::TLN-1 CRISPR + *mecDEG*) and with the TLN-1 hypomorphic allele (*ok1648*) (**Fig 5D**). 2) We were able to rescue premature termination defects caused by degradation of TLN-1 with a single copy TLN-1 transgene (**Fig 5D**). 3) We provide extensive controls for this CRISPR-based degradation system including PAT-3::GFP, UNC-112::GFP and GFP::TLN-1 alone controls and *mecDEG* alone controls (**Fig 5D**). 4) In the double mutant, we can observe the *tln-1* mutant phenotype (premature termination) during the larval stages, the RPM-1 phenotype (failed termination) does not become prevalent before adulthood (**Fig. 7**). These observations suggest that the degradation of TLN-1 is efficient in the double mutant. 5) Finally, we provide new data showing that axon termination phenotypes caused by both *rpm-1* (lf) or *mecDEG* targeting of TLN-1::GFP CRISPR are influenced by pharmacological perturbation of microtubule dynamics (**new Fig 8**). Thus, independent pharmacological approaches indicate that both RPM-1 and TLN-1 affect axon termination via differential effects on microtubule stability. This provides further mechanistic insight into why the PAT-3/UNC-112/TLN-1 adhesome axis inhibition of RPM-1 affects axon termination. This is mechanistic relationship is now summarized (**new Fig 9**).

Minor points:

1) It is interesting that TLN-1b contributes to axon termination even though it is lacking most of the C-terminal part of the protein and does not have an actin-binding domain. Have the authors tested whether re-expressing TLN-1b can rescue the phenotype observed in the absence of TLN-1?

>>>

We appreciate the reviewer’s point.

We have now performed our own sequencing of the *tln-1(ok1648)* allele, which confirms information noted on WormBase (https://wormbase.org). Our results indicate that ok1648 results in a deletion that affects TLN-1 isoform a, but does not affect the coding sequence of TLN-1 isoform b. This new point about independent verification for the ok1648 allele is included as new text in the Methods as follows:

*“We sequenced the tln-1(ok1648) allele and verified that is affects TLN-1 isoform a, c, d and e but does not affect the coding sequence for TLN-1 isoform b and f. This independently confirms what is annotated on WormBase (https://wormbase.org).”*

As the reviewer likely notes, our paper was communicated to a sister publication venue. Our new editor requested that we focus our revised manuscript around interpretation of our genetic and developmental growth cone results towards an inhibitory relationship between the adhesome and RPM-1. Thus, as requested, we focus on revising the paper extensively around this concept. Understanding which domain of TLN-1 is required for axon termination is beyond the scope of our manuscript. The reviewer’s comment about isoforms is, nonetheless, well taken and would be a good concept for future subsequent studies.

1) In Figures 4D and 5C, Chi-squared tests should be used to compare percentages. In Fig. 6F, an ANOVA is preferable for comparison of multiple observed means.

>>>

We understand the reviewer’s suggestion. Our choice of statistics for axon termination and growth cones studies is based on numerous prior published papers. We use Bonferonni correction for multiple comparisons as noted in Methods and legends. Thus, we consider our statistics valid for our genetics studies.

However, the reviewer’s comment and a point raised by another reviewer have caused us to reconsider our choice of statistics for evaluating our proteomics data. We now evaluate our proteomics data using a non-parametric Mann-Whitney test, and use a *post hoc* Benjamini-Hochberg method (*q*-value) with a 5% false discovery rate (FDR) for multiple comparisons. This is now reflected in our updated Table 1 in the following column: “Significance GS::RPM-1 versus GFP::GFP (p-value)”. Table 1 also now has p value data generated using a Mann-Whitney non-parametric test rather than a parametric Student’s t test, which was applied to normalized proteomics data. We also include a new column in Table 1: “Significance GS::RPM-1 versus GS::GFP (q-value)”. Here, we have tested our normalized proteomics data with Mann-Whitney and *post hoc* Benjamini-Hochberg method (*q*-value) with a 5% false discovery rate (FDR). To completely examine this updated analysis of our proteomics data, we direct the reviewer to updated Table 1 (including legend), updated Figure 2 (including legend), Supplemental Data File1, Supplemental Data File 2, and the statistics section of our Methods.
